# A comprehensive analysis of copy number variation in a Turkish dementia cohort

**DOI:** 10.1186/s40246-021-00346-z

**Published:** 2021-07-28

**Authors:** Nadia Dehghani, Gamze Guven, Celia Kun-Rodrigues, Catarina Gouveia, Kalina Foster, Hasmet Hanagasi, Ebba Lohmann, Bedia Samanci, Hakan Gurvit, Basar Bilgic, Jose Bras, Rita Guerreiro

**Affiliations:** 1 Department of Neurodegenerative Science, Van Andel Institute, Grand Rapids, Michigan USA; 2 Department of Genetics, Aziz Sancar Institute of Experimental Medicine, Istanbul University, Istanbul, Turkey; 3 Neuroscience Department, Michigan State University College of Natural Science, East Lansing, MI USA; 4 Behavioural Neurology and Movement Disorders Unit, Department of Neurology, Istanbul Faculty of Medicine, Istanbul University, Istanbul, Turkey; 5 Department of Neurodegenerative Diseases, Hertie Institute for Clinical Brain Research, University of Tübingen, Tübingen, Germany; 6 DZNE, German Center for Neurodegenerative Diseases, Tübingen, Germany; 7 Division of Psychiatry and Behavioral Medicine, Michigan State University College of Human Medicine, Grand Rapids, MI USA

**Keywords:** Copy number variants, Dementia, Genotyping

## Abstract

**Background:**

Copy number variants (CNVs) include deletions or multiplications spanning genomic regions. These regions vary in size and may span genes known to play a role in human diseases. As examples, duplications and triplications of *SNCA* have been shown to cause forms of Parkinson’s disease, while duplications of *APP* cause early onset Alzheimer’s disease (AD).

**Results:**

Here, we performed a systematic analysis of CNVs in a Turkish dementia cohort in order to further characterize the genetic causes of dementia in this population. One hundred twenty-four Turkish individuals, either at risk of dementia due to family history, diagnosed with mild cognitive impairment, AD, or frontotemporal dementia, were whole-genome genotyped and CNVs were detected. We integrated family analysis with a comprehensive assessment of potentially disease-associated CNVs in this Turkish dementia cohort. We also utilized both dementia and non-dementia individuals from the UK Biobank in order to further elucidate the potential role of the identified CNVs in neurodegenerative diseases.

We report CNVs overlapping the previously implicated genes *ZNF804A, SNORA70B, USP34, XPO1*, and a locus on chromosome 9 which includes a cluster of olfactory receptors and *ABCA1*. Additionally, we also describe novel CNVs potentially associated with dementia, overlapping the genes *AFG1L, SNX3, VWDE*, and *BC039545*.

**Conclusions:**

Genotyping data from understudied populations can be utilized to identify copy number variation which may contribute to dementia.

**Supplementary Information:**

The online version contains supplementary material available at 10.1186/s40246-021-00346-z.

## Background

Dementia is an umbrella term for symptoms presenting in different diseases such as Alzheimer’s disease (AD), frontotemporal dementia (FTD), and dementia with Lewy bodies (DLB). All of these diseases have significant genetic components. Mendelian early-onset Alzheimer’s disease (EOAD), for example, has been considered to be almost completely genetically determined [1]. However, causative mutations in known genes account for only a small proportion of all cases (~10% of EOAD cases), indicating that other genetic factors, still to be identified, play a substantial role in this form of AD. This missing heritability may be partly explained by genetic elements that are to-date poorly investigated and characterized, such as copy number variants (CNVs).

CNVs are segments of DNA that can have different sizes (ranging from 1 kb to several megabases) for which differences in the number of copies have been identified. These can be copy number gains (duplications or insertional transpositions), losses (deletions), gains or losses of the same locus, or multiallelic or complex rearrangements. The large size of many CNVs causes them to span or partially overlap with various genes, which may lead to unbalanced dosages of those genes. This phenomenon can lead to the development of various diseases. CNVs have been implicated in different neuropsychiatric disorders, such as autism and schizophrenia, and in neurodegenerative diseases [2]. Examples of CNVs that have been proven to cause neurodegenerative diseases include duplications of *APP* leading to EOAD [3], multiplications of alpha-synuclein (*SNCA*) causing forms of Parkinson’s disease (PD) and DLB [4], as well as both deletions and duplications of *PRKN* that are known to cause forms of PD [5].

Only a limited number of studies have assessed the role of CNVs and other structural variants in different dementias. Due to a lack of evidence for segregation, it is often not clear if these are causative, or contributing to the risk of disease. While it is understood that CNVs in known disease-associated genes play a role in the development of diseases, the effects of CNVs in other genes, with no known disease-causing mutations, can be difficult to relate to disease. A genome-wide scan of CNVs in 331 dementia cases (in which >80% of patients had a clinical diagnosis of AD), and 368 controls revealed no CNVs associated with disease using a genome-wide threshold of significance. However, the authors were able to identify a duplication of potential interest encompassing *CHRNA7*, after screening loci previously reported to be risk regions for neuropsychiatric diseases [6]. The study of 261 non-Mendelian AD families with at least one EOAD case revealed 5 deletions and 5 duplications that segregated with the phenotype. Two of these CNVs overlapped FTD genes (deletion of *CHMP2B* and duplication of *MAPT*) [7]. CNV assessments in FTD patients have revealed duplications encompassing *MAPT* [8] and a partial *GRN* deletion [9]. In two loci genome-wide associated with AD, structural variants have also been reported to be associated with disease risk: an 8-kb insertion in *CR1* that creates the CR1-S isoform harboring an extra set of C3b/C4b binding sites [10], and a 44 base-pair frameshift deletion in *ABCA7* that increases AD risk in African Americans and Caribbean Hispanics [11]. Interestingly, this latter association could be ethnic-specific as the frequency of the deletion in non-Hispanic white cohorts is much lower and has so far not resulted in significant associations with risk of disease.

There is increased awareness about the need to represent diverse populations in genetic studies (for a recent review related to AD genetics see [12]). Such contributions include our previous studies of a Turkish dementia cohort where we described mutations in the known Mendelian AD genes [13], a *NOTCH3* variant, known to be causative for cerebral autosomal dominant arteriopathy with subcortical infarcts and leukoencephalopathy, in an AD case [14], three homozygous *TREM2* variants and a compound heterozygous variant in FTD-like families [15, 16], and no variants or CNVs in *TYROBP*/DAP12, which encodes a TREM2 ligand [17]. Additionally, this cohort has been screened for variants in genes causative for FTD [18]. We previously reported a preliminary analysis of copy number variation in a subset of this cohort as a poster presentation [19]. Here, we complement this work by assessing the potential role of CNVs in the etiology of dementia in this population.

## Results

### Family analysis

**Fig. 1:**
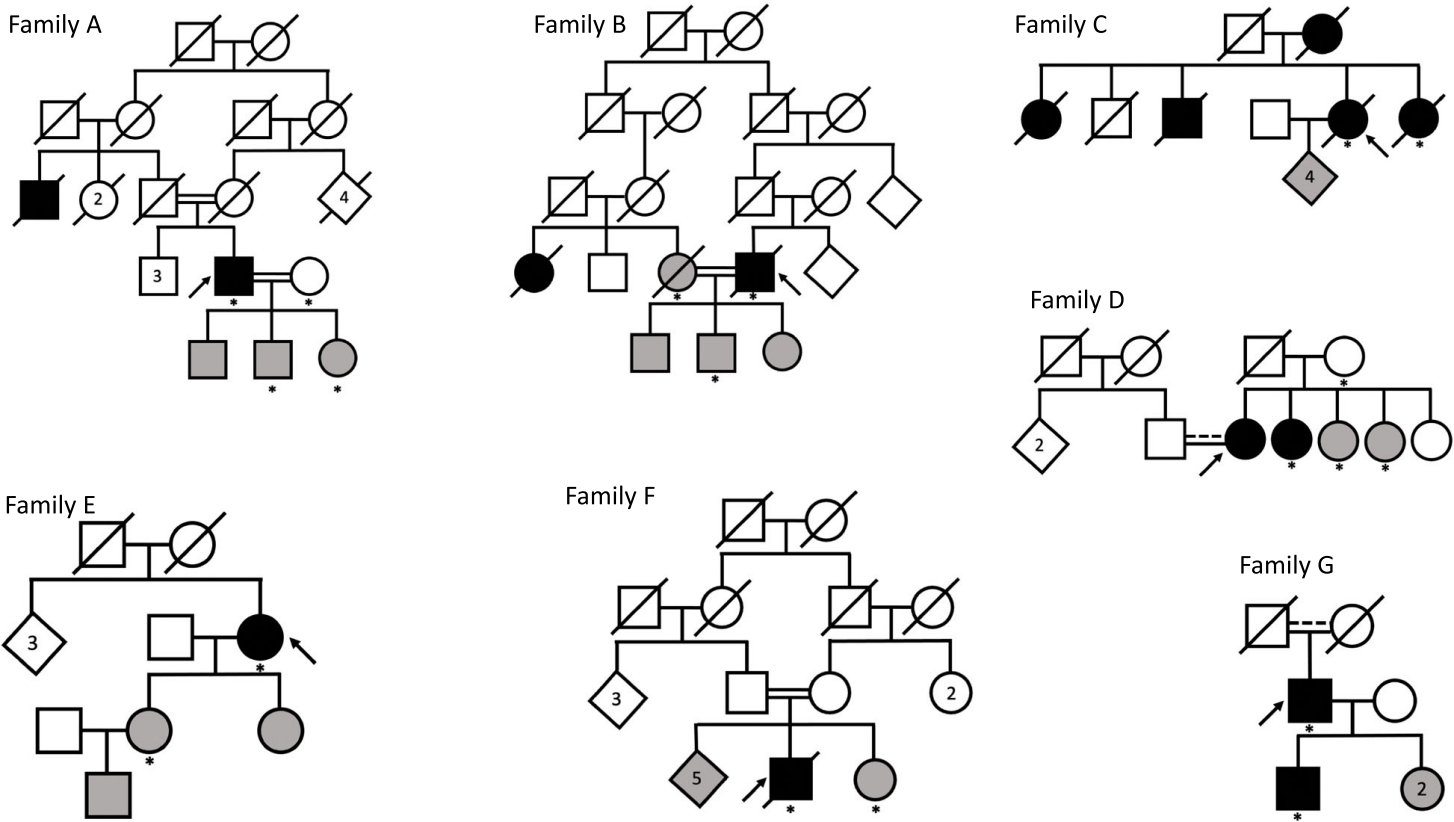
Pedigree trees for the seven families studied. Squares represent males, circles represent females, and diamonds represent any sex, with numbers representing more than one individual. Diagonal lines indicate that the individual is deceased. Double horizontal bars represent consanguineous marriages and a dotted line suggests consanguinity without support from the full pedigree tree. Black fill indicates affected status, white indicates unaffected and gray indicates risk. Family members genotyped in this study are marked with an asterisk (*). Arrows indicate probands

CNVs were called in trios or quartets for seven Turkish families to analyze segregation of CNVs; pedigree trees are illustrated in Fig. 1.

The sibling pair from family C were both affected with dementia, with the proband receiving a diagnosis of FTD. The proband and three offspring carry a *GRN* variant ENST00000053867.3:c.415T>C, which co-segregates with a *GRN* variant ENST00000053867.3:c.-22C>T; both of these variants were previously reported with unclear pathogenicity [18]. The proband died aged 77 years, and the age at last examination was 74 years for the affected sibling genotyped. One duplication overlapping *AFG1L* and *SNX3* was found to be shared by the siblings (Supplementary Fig. 1). Subsequent assessment of this candidate CNV in the entire Turkish dementia cohort revealed no other CNVs in the same locus. There was also no overlap with CNVs called in individuals from the UK Biobank (UKBB).

In family G, where both father and son were diagnosed with FTD (ages at onset 77 and 45 years, respectively), a shared duplication was detected overlapping *VWDE*. This CNV intersected with a duplication in 1 non-dementia and zero dementia individuals from the UKBB.

Following our filtering criteria, no candidate CNVs were identified in families A, B, D, E, and F. Of these, only family A was a real quartet, with both parents and two offspring genotyped. A probably pathogenic *NOTCH3* variant was previously reported in both the father diagnosed with AD (age at onset 62 years) and one offspring who was not genotyped [14]. Ages at last examination were 74, 73, 51, and 50 years for father, mother, son, and daughter, respectively.

From family B, we genotyped a trio with consanguineous parents including the father diagnosed with FTD (age at onset 82 years), and both the mother and son at risk of developing disease, with ages at last examination for the latter being 79 and 49 years, respectively. Based on relatedness between parents, as indicated on the pedigree tree (identity-by-descent 0.1048), mother-father segregation of CNVs was possible and taken into account in the analysis.

In family D, the proband was diagnosed with FTD aged 39 years, the mother was unaffected at last examination aged 79 years, and the additionally genotyped siblings were 37 and 34 years at last examination.

From family E, we genotyped the mother diagnosed with FTD (age at onset 51 years) and daughter at risk (36 years at last examination).

The two siblings in family F were previously reported to carry a *PSEN2* variant ENST00000366783.3:c.520A>G reported as probably pathogenic [13], although another report suggested this is likely non-pathogenic [20]. The proband was diagnosed with FTD at 52 years, whilst at last examination, the additionally genotyped sibling was 36 years.

Table 1 summarizes the candidate CNVs identified from this analysis in families C and G.

**Table 1:** Results from the analysis of CNVs in 7 Turkish families

Family	Position	# SNPs	Length (bases)	CN	Family members with CNV	GnomAD-SV spanning the entire CNV?	Gene	Total # intersects with CNVs from UKBB dementia (CN=0|CN=1|CN=3|CN=4)	Total # intersects with CNVs from UKBB non-dementia (CN=0|CN=1|CN=3|CN=4)	Pathogenicity
C	Chr6:108570894-108694389	15	123,496	3	Affected siblings	Inversions in 2	*AFG1L, SNX3*	No overlap	No overlap	Uncertain significance
G	Chr7:12371801-12439672	35	67,872	3	Affected father and affected son	Loss of function deletion in 1	*VWDE*	No overlap	1 (0|0|1|0)	Uncertain significance

*CN* copy number, *0﻿* homozygous deletion, *1﻿* heterozygous deletion, *3﻿* duplication, *﻿4* triplication. No UKBB CNVs were significantly associated with dementia based on Fisher’s exact test. Neither of these candidate CNVs intersected CNVs detected in the broader Turkish cohort

### CNVs in neurogenes or previously reported in neurodegenerative diseases

We next sought to determine if CNVs overlapping well-established neurodegenerative disease genes, or genes implicated in similar analyses were present in this Turkish dementia cohort. Some previously reported findings, such as *CR1* duplications, could not be confirmed with our data due to a lack of coverage or resolution at the low copy repeat regions.

As summarized in Table 2, there were CNVs which were both not common in the Database of Genomic Variants (DGV) and had no evidence to refute their association with dementia in the UKBB. Such CNVs included a duplication overlapping *ZNF804A* detected in one Turkish AD patient; heterozygous deletions in a region overlapping this gene have been previously reported in 2 AD cases, 4 mild cognitive impairment (MCI) cases, and one control [21].

**Table 2:** Results of intersecting CNVs detected in the Turkish dementia cohort and both neurodegenerative disease-associated genes and genes previously reported in CNV analyses of similar disease cohorts

Position	# SNPs	Length (bases)	CN	Sample	GnomAD-SV spanning the entire CNV?	# DGV observations	Genes (neuro/lit gene)	Total # intersects with CNVs from UKBB dementia (CN=0|CN=1|CN=3|CN=4)	Total # intersects with CNVs from UKBB non-dementia (CN=0|CN=1|CN=3|CN=4)	Pathogenicity
Chr1:12850542-12915847	16	65,306	3	F ;AD; AAO: 72	No	309	*PRAMEF1, PRAMEF11,* ***HNRNPCL1***	21 (0|9|12|0)	46(0|19|27|0)	Likely benign
Chr15:22750305-23226254	113	475,950	3	F ;FTD	Loss of function deletion in 41	452	*TUBGCP5,* ***CYFIP1****, NIPA2,* ***NIPA1***	22 (0|9|13|0)	44(0|16|28|0)	Likely benign
				M; AD; AAO: 64						
Chr2:185421477-185564834	24	143,358	3	M ;AD ;AAO: 71	Inversion in 1	4	***ZNF804A***	No overlap	No overlap	Uncertain significance
Chr2:61448493-61929733	59	481,241	3	F; AD; AAO: 52	No	0	***SNORA70B, USP34, XPO1***	No overlap	No overlap	Uncertain significance
Chr22:18886915-19037734	59	150,820	3	M; AD; AAO: 77	No	50	*DGCR6, PRODH,* ***DGCR2***	41 (0|8|33|0)	67(0|8|59|0)	Uncertain significance
Chr3:1154559-2400967	605	1,246,409	3	M; MCI ;AAO: 73	No	1	***CNTN6****, CNTN4*	11 (0|7|4|0)	20(0|7|13|0)	Uncertain significance
Chr4:172006196-172792316	140	786,121	3	F; AD; AAO: 75	No	0	***GALNTL6***	1 (0|1|0|0)	2(0|2|0|0)	Uncertain significance
Chr6:31360254-31451679	161	91,426	1	F; RISK	No	657	***MICA***	7 (0|5|2|0)	17(0|10|7|0)	Likely benign
Chr6:31360254-31454364	168	94,111	3	F; AD; AAO: 77	No	122	***MICA***	7 (0|5|2|0)	17(0|10|7|0)	Uncertain significance
				M; AD; AAO: 65						
Chr6:4805260-5449240	201	643,981	3	M; MCI; AAO: 73	No	0	***MIR3691****, CDYL, RPP40, PPP1R3G, LYRM4, FARS2*	1 (0|1|0|0)	1(0|1|0|0)	Uncertain significance
Chr9:107311368-107699196	246	387,829	1	M; MCI; AAO: 73	Copy gain in 1	1	***OR13C8, OR13C5, OR13C2, OR13C9, OR13D1, NIPSNAP3A, NIPSNAP3B, ABCA1, LOC286367***	1 (0|0|1|0)	No overlap	Uncertain significance

*M* male, *F* female, *AD* Alzheimer’s disease, *FTD* Frontotemporal dementia, *MCI* mild cognitive impairment, *AAO* age at onset, *CN* copy number, *0* homozygous deletion, *1* heterozygous deletion, *3* duplication, *4* triplication. Neurogenes and genes previously reported are in bold lettering. No UKBB CNVs were significantly associated with dementia based on Fisher’s exact test

We also detected a duplication overlapping *SNORA70B, USP34*, *and XPO1* in one AD patient; a deletion has been previously reported in an MCI patient [21].

We observed a duplication overlapping *GALNTL6* in one AD patient. A previous study reported an association between *GALNTL6* CNVs and AD AAO; the significance of this association was lost when corrected for *APOE*; however, the authors mentioned that *GALNTL6* had been previously associated with lipid metabolism, body mass index, and hypertension, suggesting a potential association with AD through a vascular mechanism [22]. Heterozygous deletions intersecting our identified CNV were detected in 1 dementia and 2 non-dementia individuals from the UKBB.

We detected a duplication in one Turkish MCI case overlapping *MIR3691*; a duplication overlapping this microRNA was reported in one French early-onset AD case [23]. In the UKBB, 1 dementia and 1 non-dementia individual carried heterozygous deletions intersecting this region.

**Fig. 2:**
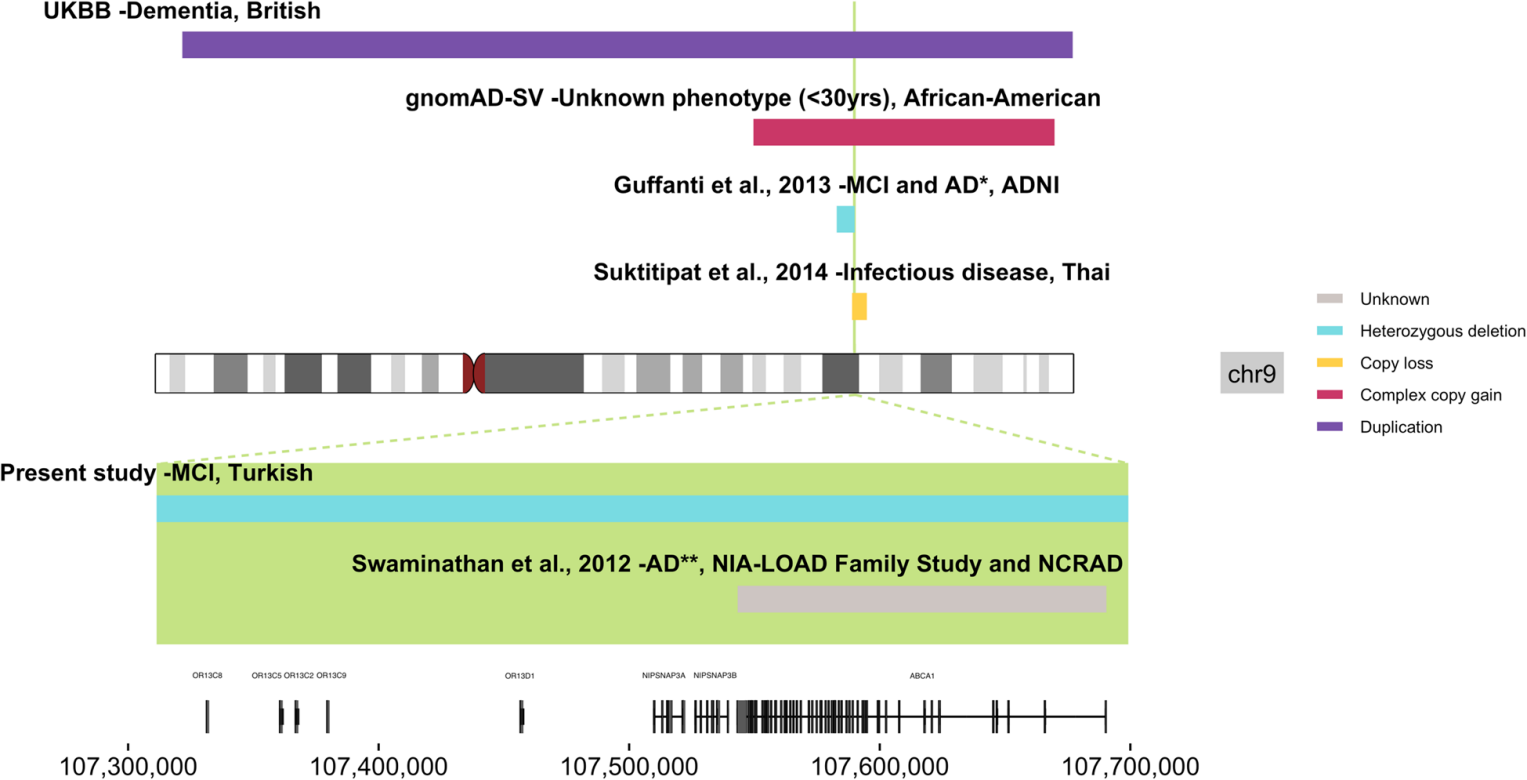
Plot for CNVs overlapping *ABCA1* and a cluster of olfactory receptor genes on chromosome 9. The Turkish CNV region is highlighted in green, with the longest transcripts for the overlapping genes plotted below. CNVs extending beyond the Turkish CNV are plotted above the chromosome. AD= Alzheimer’s disease, ADNI= Alzheimer’s Disease Neuroimaging Initiative, gnomAD-SV= genome aggregation database structural variants, MCI= mild cognitive impairment, NCRAD= National Centralized Repository for Alzheimer’s Disease and Related Dementias, NIA-LOAD= National Institute on Aging- late onset Alzheimer’s disease, UKBB= UK Biobank. *This CNV region encompasses heterozygous deletions reported in 1 MCI patient and 4 AD patients. **This CNV was reported in 3 AD patients.

We also observed a heterozygous deletion overlapping *ABCA1, LOC286367, NIPSNAP3A, NIPSNAP3B, OR13C2, OR13C5, OR13C8, OR13C9*, and *OR13D1* in one MCI patient. Heterozygous deletions in a CNV region overlapping these genes have been previously reported in 1 MCI and 4 AD cases [21]. CNVs of unreported type overlapping *ABCA1* were described in 3 AD participants and no controls by Swaminathan and colleagues [24]. Although olfactory receptor genes frequently harbor CNVs [25], the heterozygous deletion spanning this region was not considered common in the genome aggregation database structural variants (gnomAD-SV). From the UKBB, only one dementia individual harbored a duplication intersecting this region. Additionally, one deletion spanning this region was reported in DGV, in a Thai infectious disease cohort [26]; these CNVs are collectively illustrated in Fig. 2. We observed instances of copy number discordance for CNVs overlapping the same loci. Potential considerations when interpreting such results include differences in cohort sizes, populations, and diagnoses.

Moreover, we observed CNVs that we defined as common on DGV or report evidence suggesting a lack of association with dementia in the UKBB. One such CNVs was a duplication overlapping *HNRNPCL1* detected in an AD patient; CNVs of unreported type overlapping this gene were previously described in 6 AD cases, 4 MCI cases, and zero healthy controls [27].

We detected a duplication overlapping both *CYFIP1* and *NIPA1* in one AD and one FTD patient. A similar duplication was previously reported in 10 Caribbean Hispanic AD cases and 3 controls, with dosage increase of *CYFIP1* and *NIPA1* genes confirmed by quantitative PCR [28].

A duplication overlapping *DGCR2* was identified in only the affected father of family G. Although this was not common on DGV, there was no significant association with dementia in the UKBB. CNVs of unreported type overlapping this gene have been previously reported in at least 4 AD cases and no controls [29]. This CNV also overlaps *PRODH*; this encodes proline dehydrogenase which is involved in a key step before the production of glutamate. Elevated proline levels have been previously associated with schizophrenia and bipolar disorder [30], and this has been proposed to be investigated in AD.

We observed a duplication overlapping *CNTN6* in one MCI patient; two duplications overlapping this gene were previously reported in a Caribbean Hispanic AD study [28].

We report one heterozygous deletion and two duplications spanning *MICA*; to note, the heterozygous deletion overlapping *HCG26*, *HCP5*, *MICA*, and *PMSP* was detected in the to-date unaffected sibling in family F (no CNV overlapping this gene was detected in the affected sibling). This was not reported in the family analysis since deletions (and for that matter, duplications) spanning this region were common on DGV. Moreover, CNVs intersecting this region were not significantly associated with dementia in the UKBB. CNVs of unreported type overlapping this gene were previously reported in four AD patients (and no controls) [24].

### CNVs shared within the cohort

Although many candidate CNVs were identified in previous analyses in our study, our approach to test for shared CNVs enabled us to identify additional, potentially novel, CNVs associated with dementia in our cohort, as summarized in Table 3. There were no additional reports in the literature on *BC039545*, where we observed overlapping deletions in two Turkish FTD patients.

**Table 3:** Results of shared gene analysis for CNVs with same copy number

Shared genes	CN	Sample (#|diagnosis)	GnomAD-SV entirely spanning at least one CNV?	# DGV observations	Total # intersects with CNVs from UKBB dementia (CN=0|CN=1|CN=3|CN=4)	Total # intersects with CNVs from UKBB non-dementia (CN=0|CN=1|CN=3|CN=4)	Pathogenicity
*BC039545*	1	2|FTD	No	0	No overlap	No overlap	Uncertain significance
AFG1L, SNX3*	3	1|FTD 1|dementia	Yes	No overlap	No overlap	No overlap	Uncertain significance

*FTD* frontotemporal dementia, *CN* copy number, *0* homozygous deletion, *1* heterozygous deletion, *3* duplication, *4* triplication. See Supplementary Table 1 for details of exact CNVs and sample diagnoses. Asterisk (*) indicates a CNV also reported in our family analysis. No UKBB CNVs were significantly associated with dementia based on Fisher’s exact test

## Discussion

This is the first study reporting CNVs in a Turkish dementia cohort. We report novel CNVs that may be causing or be associated with disease, replicate some CNVs previously reported in other dementia cohorts, and utilize a larger dataset of both dementia and non-dementia individuals from the UKBB to further investigate the potential association of candidate CNVs with the studied phenotypes. When comparing CNVs observed in this Turkish cohort to those detected in the UKBB dementia individuals, we observed both CNVs with the same and opposite dosage. The latter is perhaps a more frequent phenomenon than expected, where both deletions and duplications are observed at over 99% of copy number variant regions [31].

From our family analysis, we report potentially novel duplications associated with disease overlapping the genes *AFG1L, SNX3*, and *VWDE*. Deletions overlapping *BC039545* were additional findings from our analysis of shared genes. As expected, due to lack of previous reports, most CNVs are of uncertain significance. *AFG1L* encodes a mitochondrial membrane protein and increased protein expression has been shown to induce apoptosis [32]. Downregulated *AFG1L* expression (also known as *LACE1*) was reported in AD patients compared to controls [33]. Variants in genes that regulate retromer function, including *SNX3*, have been associated with AD [34]. Moreover, there is evidence to suggest that SNX3 regulates amyloid-beta production [35]. Retromer dysfunction has been implicated in neurodegenerative diseases including FTD [36]. This duplication, together with the previously reported *GRN* variants, may increase the genetic risk of FTD. *VWDE* encodes Von Willebrand Factor D and EGF Domains. This locus has been previously identified as a potential modifier of disease risk in a genome-wide association study of FTD patients with *GRN* mutations [37].

Our analysis of CNVs overlapping neurogenes and genes implicated in previous CNV studies of similar cohorts contributes further evidence for understanding the potential involvement of such events in disease. One example of a region with conflicting reports overlaps *CYFIP1* and *NIPA1* where we observed duplications in two Turkish dementia patients. Since previous reports identified *CYFIP1* and *NIPA1* duplications in three unaffected individuals, CNVs in these genes are most likely not causative for AD; however, potential associations with increased risk of AD were considered [28]. *CYFIP1* is linked with the *APP* translation repressor, FMRP, suggesting that this CNV may alter *APP* turnover. *CYFIP1* deletions have been reported in CNV analyses of schizophrenia [38]. Our candidate CNVs overlapping both *CYFIP1* and *NIPA1* were predicted as likely benign by ClassifyCNV, and we did not observe a significant association with such CNVs and dementia in the UKBB. However, these duplications in Turkish individuals had a positive likelihood ratio above 1 (Supplementary Table 2).

Deletions spanning a cluster of olfactory receptors on chromosome 9 may increase susceptibility to dementia since there are no structural variants in gnomAD-SV spanning all genes in this cluster; this is interesting considering that olfactory dysfunction is a common prodromal symptom of neurodegenerative diseases [39]. Increased copy number encompassing a different cluster of olfactory receptors was previously associated with earlier AD age at onset; we did not detect any overlap between these genes and CNVs from our broader dementia cohort [40]. The *ABCA1* gene, also impacted by these deletions, is a cholesterol transporter from the ATP-binding cassette transporter superfamily. Mutations in this gene cause Tangier disease which is characterized by reduced levels of plasma high density lipoproteins (HDL) [41]. Intriguingly, the long noncoding RNA LOC286367 has been implicated in mediation of ABCA1 expression, at least in the context of propofol treatment [42]. In a recent AD GWAS, rs1800978 in *ABCA1* was identified as the lead SNP in a new genome-wide significant locus [43]. Together with the vasoprotective functions of HDL and our observed deletion spanning this gene in an MCI patient, there is increasing evidence suggesting a role of *ABCA1* in AD [44]. Also located in this CNV region, *NIPSNAP3A* gene expression in white matter has been significantly negatively correlated with neurocognitive impairment [45].

We identified a duplication overlapping *ZNF804A* in one AD patient*.* Heterozygous deletions overlapping this gene were previously reported in 2 AD cases, 4 MCI cases, and one control [21]. The gene encoding zinc finger binding protein *ZNF804A* is a well-recognized schizophrenia risk gene knockdown of which has been shown to decrease TYROBP protein expression in a variant CNS catecholaminergic cell line [46, 47]. Variants in *TYROBP*, encoding an adaptor protein for TREM2, have been reported in EOAD patients [48].

We also detected a duplication overlapping *SNORA70B, USP34*, and *XPO1* in one AD patient; a deletion has been previously reported in an MCI patient [21]. *USP34* encodes a ubiquitin-specific protease contributing to the prevention of proteasomal degradation of proteins. USP34 expression was downregulated in hippocampi of patients with AD compared to controls [49]. *USP34* has been identified as a *YAP1*-regulated gene; *YAP1* has been proposed as an upstream regulator of AD development [50]. More recently, *Usp34* was identified as a differentially expressed gene between lipid-droplet-low and lipid-droplet-rich microglia from aged mice with such changes perhaps contributing to impaired phagocytosis [51]. *XPO1* encodes exportin 1 which is involved in the nuclear export of proteins. *XPO1* has been reported to modify amyloid-beta toxicity; however, reports for the association of *XPO1* variants with AD were not conclusive [52, 53]. Inhibition of XPO1 is not sufficient to promote nuclear localization of TDP-43 and FUS; two proteins which accumulate in the cytoplasm in ALS and FTD [54–56]. Potential mechanisms by which XPO1 inhibition may prevent neurodegeneration is by modulating autophagy [57]. Haploinsufficiency of *USP34* and *XPO1* has been postulated as a mechanism of disease in a patient with mild intellectual disability and cranio-facial dysmorphisms [58].

Limitations of this study include both low sample size and the absence of Turkish controls to cross-reference with and propose new CNVs. However, we utilize data from both dementia and non-dementia UKBB individuals in order to conduct this comparison. Additionally, novel small CNVs are difficult to identify using genotyping data, particularly when the intensity of the probes frequently deviates from the normalized value. To minimize this issue, we used best practices to make the CNV calls; still, there is the caveat that small CNVs may be missed.

There is a clear lack of studies utilizing whole genome genotyping data to detect CNVs in Alzheimer’s disease. This type of data is widely available in the AD field, so there is the ability to conduct similar CNV analyses in much larger datasets to unequivocally replicate existing candidate CNVs and identify new ones. Additionally, large datasets with raw genotyping data available for download, such as the UKBB, are particularly powerful resources, although it should be noted that this dataset is predominantly composed of individuals with European ancestry.

## Conclusions

Our analysis of CNVs in a Turkish dementia cohort extends previous reports of CNVs overlapping the genes *ZNF804A, SNORA70B, USP34, XPO1,* and a locus on chromosome 9 which includes a cluster of olfactory receptors and *ABCA1.* With regard to *ZNF804A,* both duplications and deletions have now been reported in AD. The duplication spanning *SNORA70B, USP34*, and *XPO1* in a Turkish AD patient contrasts the deletion previously reported in MCI and previous molecular studies implicating these genes in neurodegeneration. Our report of a Turkish MCI patient with a deletion spanning *ABCA1* replicates previous reports in both AD and MCI and increases evidence for a role of such CNVs in disease. We also report novel duplications, potentially associated with FTD, overlapping the genes *AFG1L, SNX3,* and *VWDE* from our family analyses. Deletions overlapping *BC039545* in two Turkish FTD patients were additional findings from our analysis of shared genes. Future studies in both larger cohorts (including extended family members and new families demonstrating segregation of these CNVs) and additional populations are necessary to replicate these findings for better understanding of their contributions to disease. The detailed genetic study of this cohort has led to identifying several genetic factors associated with dementia in the Turkish population. The current study adds CNVs to the variants with potential roles in different forms of dementia in this population. In addition, given the degree of consanguinity observed in the cohort, we are also systematically assessing the role of variants within extended runs of homozygosity to characterize the genetics of dementia in the Turkish population completely.

## Methods

### Cohort

We studied a Turkish dementia cohort of one hundred twenty-four individuals with either MCI, AD, FTD, or at risk of such diseases due to family history and age below the age of disease onset. All participants were recruited at the Behavioral Neurology and Movement Disorders Unit outpatient clinic of Istanbul Faculty of Medicine, Istanbul University, and underwent detailed clinical and neuropsychological examination and, if possible, cerebral magnetic resonance imaging (MRI) or positron emission tomography (PET) imaging. Diagnosis of dementia was based on the National Institute of Neurological and Communicative Disorders and Stroke and the Alzheimer’s Disease and Related Disorders Association (NINCDS/ADRDA) [59], FTD was defined following the Lund and Manchester criteria [60], and MCI patients were diagnosed as described by Frank and Petersen [61]. The individuals selected to be genotyped were commonly probands of affected families. Descriptions of cohort phenotypes are in Table 4. Since CNVs can also be involved in phenotype modulation, we included individuals who have had variants previously reported, pathogenic, or otherwise, as listed in Supplementary Table 3. DNA was extracted from whole blood using standard procedures. The study was approved by the Ethics Committee of Istanbul Faculty of Medicine, Istanbul University. A neurologist took the necessary clinical information after obtaining informed written consent from the patients and their participating family members. Consent was provided by the legally authorized representative for subjects unable to consent.

**Table 4:** Description of cohort phenotypes, number of individuals for each sex and mean age at onset, and last clinic visit for all individuals studied (n=124)

Phenotype	Total # individuals (M|F)	Mean age at onset (±SD)	Mean age at last clinic visit (±SD)
MCI	22 (13|9)	70.0 (±7.7)	74.0 (±7.0)
AD	58 (20|38)	65.1 (±8.8)	69.8 (±10.0)
FTD	27 (15|12)	56.5 (±11.1)	56.4 (±15.9)
Dementia	1 (0|1)	NA	74.0 (±0)
Risk of AD	4 (3|1)	NA	53.3 (±1.4)
Risk of FTD	10 (3|7)	NA	47.3 (±17.1)
Unaffected	2 (0|2)	NA	72.0 (±1.4)

*MCI* mild cognitive impairment, *AD* Alzheimer’s disease, *FTD* frontotemporal dementia, *F* female, *M* male, *SD* standard deviation, *NA* not available. This cohort includes 19 individuals analyzed as part of seven families (Fig. 1).

### Genotyping

One hundred twenty-four Turkish individuals were genotyped using Illumina’s HumanOmniExpress arrays (Illumina, Inc., CA, USA). Intensity files were analyzed using GenomeStudio (GS) v2.0.4 software (Illumina, Inc., CA, USA) along with the respective manufacturer’s cluster files. Quality control (QC) procedures were performed in GS prior to CNV analysis as described by Jarick and colleagues, including exclusion of SNPs with a GenTrain score below 0.7 [62]. Five genotyped individuals (2 male MCI, 1 female AD, 2 FTD (1 male, 1 female)) were not analyzed for CNVs based on call rate <0.97 or low-quality raw data upon visualization in GS. Therefore, CNVs called from 119 individuals were used in downstream analyses.

### Calling CNVs

Copy number variants in autosomes were called using signal intensity files exported by GS, with PennCNV-1.0.5 software [63] (positions in hg19). PennCNV analysis was performed using a PFB file for Human OmniExpress arrays from www.penncnv.openbioinformatics.org, updated on August 16, 2011, and a GC-model adjustment, as per the recommended protocol. Automatic quality control of CNV calls was not used with PennCNV since individuals with low-quality raw data were manually excluded before the analysis. CNVs were filtered to consider those with at least 10 SNPs and at least 50kb in length. Adjacent CNVs were merged based on the default parameters, and CNVs were excluded if they were overlapping telomeres, centromeres, known segmental duplications, and the immunoglobulin, or T cell receptor loci. The final step of the PennCNV analysis involved identifying genes overlapping the CNV calls. Results were further filtered based on complete overlap with CNVs of the same copy number reported in the genome aggregation database structural variants (gnomAD-SV) [64] and part of the curated NCBI Common Structural Variants track which uses the following criteria to define common structural variants: >50bp, variants occurring in at least 100 individuals, allele frequency ≥0.01. The 2020-02-25 release of the Database of Genomic Variants (DGV) was utilized in a similar manner; Turkish CNVs with complete overlap by CNVs of the same type reported in at least 100 individuals in DGV were filtered [65]. Candidate CNVs were visually inspected with GS using cnvPartition v3.2.1 plug-in (Illumina, Inc). In order to determine the presence and copy number of a CNV, measurements of the log R ratio and the B allele frequency were utilized.

### CNVs segregating within families

This cohort included seven families; therefore, we first conducted a genome-wide scan for CNVs that segregated with disease within each of these families. We identified families based on information from pedigree trees (Fig. 1). We also calculated identity-by-descent to confirm relatedness within families; based on the same measure, there was no relationship between the 7 families described.

We specifically re-called CNVs in family trios or quartets using PennCNV. The CNV had to be present in at least 1 affected individual and absent in unaffected family members, or only present in unaffected family members which would suggest a protective effect. Family analyses were not conducted on two families with definitely pathogenic variants reported in *TREM2.* These analyses were only conducted in families where either no variants were identified to be the cause of disease, or in families where variants were identified, but these were of unknown significance for the disease. Candidate CNVs resulting from these family analyses were subsequently assessed in the entire Turkish dementia cohort.

### Overlap with “neurogenes” and genes previously reported in CNV analyses of similar dementia cohorts

We assessed our CNV data from PennCNV for CNVs overlapping specific genes known to be related to neurodegenerative diseases [66]; we refer to these as “neurogenes” (Supplementary Table 4).

Additionally, we conducted a literature search for CNVs previously reported in dementia, MCI, AD, or FTD and compiled the respective list of genes (Supplementary Table 5). We combined this list with the “neurogenes” list to obtain a final set of genes (all in autosomes) for which overlapping CNVs were specifically assessed.

### CNVs shared within the cohort

We searched for CNVs of the same copy number overlapping the same genes, present in two or more individuals of the studied cohort. Results were further filtered to remove CNVs that were completely localized within 2Mb of autosome ends, based on reported UCSC positions (hg19).

### Intersection with dementia and non-dementia individuals from the UK Biobank (UKBB)

We used data from the UK Biobank to validate results. For this, CEL files from 2594 and 4570 UK Biobank dementia or non-dementia individuals, respectively, were downloaded and CNVs were called using both Affymetrix Power Tools [67], and PennCNV, as described above. There were two quality control (QC) steps. The first QC step utilized dish QC (DQC) values which are based on intensities of probe sequences for non-polymorphic genome locations. A DQC value of zero indicates no resolution between the distributions of AT and GC probe contrast values, and a DQC value of 1 indicates perfect resolution. Individuals with a DQC value less than the default threshold of 0.82 were filtered out. The second QC step filtered out individuals with a QC call rate value less than the default threshold of 97%. Following QC, CNVs were detected from 2323 and 4567 UK Biobank individuals with dementia or non-dementia, respectively (hg19). All individuals were actively enrolled in the UKBB study. Exact positions of candidate CNVs from the three analyses were intersected with the CNVs called for UKBB individuals; for the analysis of shared CNVs, the largest encompassing region was used for the intersect. Fisher’s exact test (two-sided) was performed to compare the detection of CNVs overlapping candidate loci between UKBB dementia and non-dementia individuals. When reporting novel dementia CNVs, results were considered significant if *p*<0.05.

### Prediction of CNV pathogenicity and likelihood ratios

We assessed CNV pathogenicity using ClassifyCNV [68]. This tool implements the 2019 American College of Medical Genetics and Genomics classification guidelines [69]. We also report values from likelihood ratio tests for both CNVs reported in Turkish individuals, and overlapping UKBB CNVs with the same copy number. Positive likelihood ratio = $\frac{\frac{TP}{TP+ FN}}{\frac{FP}{FP+ TN}}$ and negative likelihood ratio = $\frac{\frac{FN}{TP+ FN}}{\frac{TN}{FP+ TN}}$, where TP = true positive, FN = false negative, TN = true negative, and FP = false positive.

## Supplementary Information

**Additional file 1: Supplementary Figure 1**. GS plot illustrating duplications spanning AFGL1/LACE1 and SNX3 in a sibling pair from family C.

**Additional file 2: Supplementary Table 1**. Shared CNVs of the same copy number spanning the same genes in two or more individuals.

**Additional file 3: Supplementary Table 2**. Likelihood ratios for CNVs reported from analysis of both Turkish dementia cohort and UK Biobank.

**Additional file 4: Supplementary Table 3**. Previously reported definitely and potentially pathogenic variants in individuals included in this cohort.

**Additional file 5: Supplementary Table 4**. List of neurogenes.

**Additional file 6: Supplementary Table 5**. Genes reported to overlap CNVs associated with dementia.

## Data Availability

The datasets used and/or analyzed during the current study are available from the corresponding author on reasonable request.

## Abbreviations

CNVs: Copy number variants
AD: Alzheimer’s disease
FTD: Frontotemporal dementia
DLB: Dementia with Lewy bodies
EOAD: Early-onset Alzheimer’s disease
SNCA: Alpha-synuclein
PD: Parkinson’s disease
MCI: Mild cognitive impairment
MRI: Magnetic resonance imaging
PET: Positron emission tomography
NINCDS/ADRDA: National Institute of Neurological and Communicative Disorders and Stroke and the Alzheimer’s Disease and Related Disorders Association
GS: GenomeStudio
QC: Quality control
gnomAD-SV: Genome aggregation database structural variants
DGV: Database of Genomic Variants
UKBB: UK Biobank
DQC: Dish QC
HDL: High-density lipoproteins
