# Supplementary figures and images for: A comprehensive analysis of copy number variation in a Turkish dementia cohort
Source: Hum Genomics. 2021 Jul 28;15:48. doi: 10.1186/s40246-021-00346-z (PMC8317312; doi:10.1186/s40246-021-00346-z)

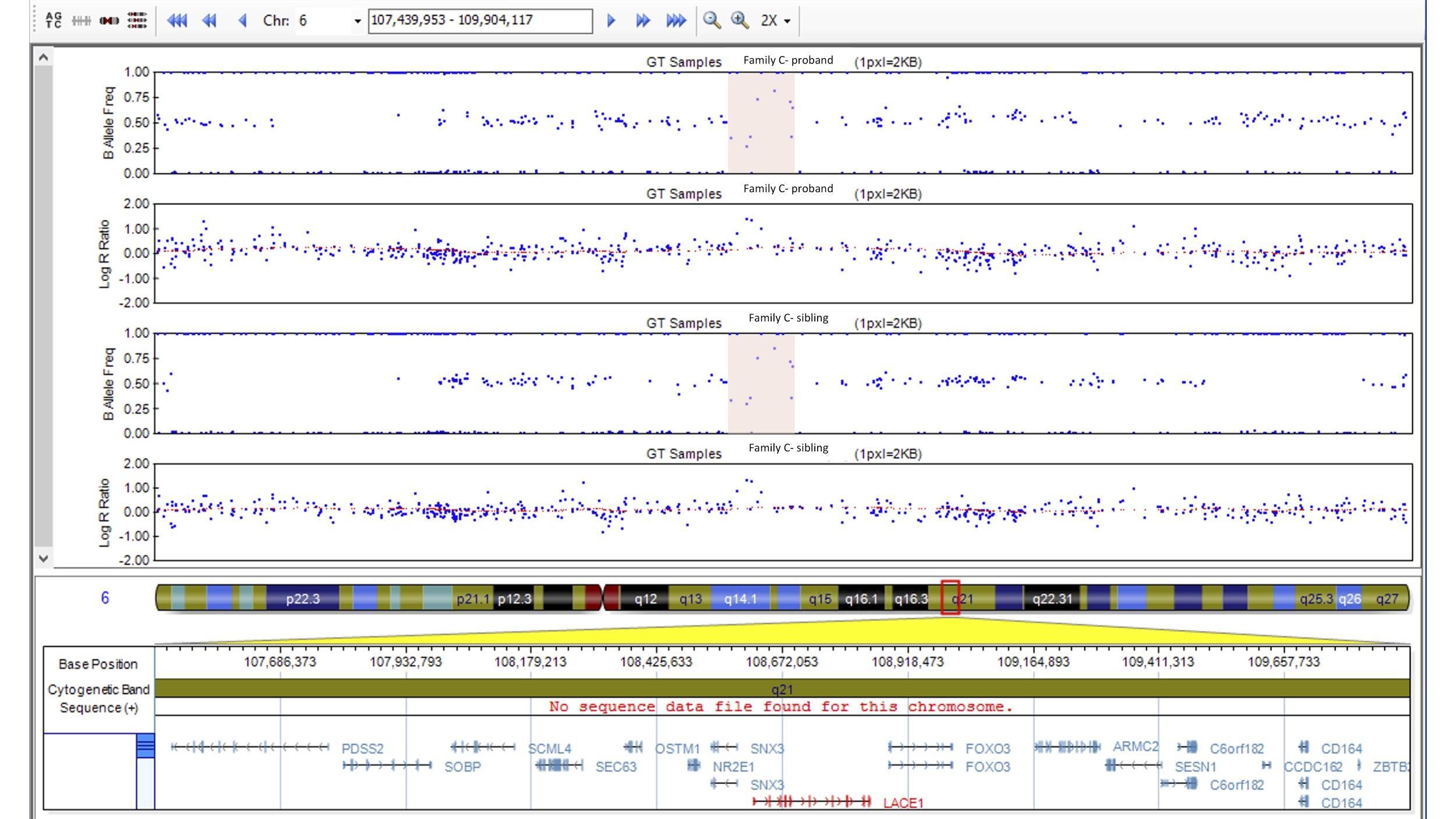

Supplement: Supplementary file 1 — Additional file 1: Supplementary Figure 1. GS plot illustrating duplications spanning AFGL1/LACE1 and SNX3 in a sibling pair from family C. [file 40246_2021_346_MOESM1_ESM.docx]
