# Supplementary material for: A comprehensive analysis of copy number variation in a Turkish dementia cohort
Source: Hum Genomics. 2021 Jul 28;15:48. doi: 10.1186/s40246-021-00346-z (PMC8317312; doi:10.1186/s40246-021-00346-z)
Supplement: Supplementary file 2 — Additional file 2: Supplementary Table 1. Shared CNVs of the same copy number spanning the same genes in two or more individuals. [file 40246_2021_346_MOESM2_ESM.docx]

| **Position** | **# SNPs** | **Length (bases)** | **CN** | **Diagnosis** | **GnomAD-SV spanning the entire CNV?** | Gene (**shared**) |
| --- | --- | --- | --- | --- | --- | --- |
| Chr15:38258772-38329343 | 16 | 70,572 | 1 | F; FTD; AAO: 72 | No | ***BC039545*** |
| Chr15:38270156-38326720 | 11 | 56,565 |  | F; FTD; age: 37 |  |  |
| Chr6:108570894-108694389 | 15 | 123,496 | 3 | F; FTD; age: 74 | Inversion in 2 | ***AFG1L, SNX3*** |
| Chr6:108570894-108713146 | 17 | 142,253 |  | F; FTD; age: 73 |  |  |

M= male, F= female, FTD= Frontotemporal dementia, AAO= age at onset, CN= copy number, 1= heterozygous deletion, 3= duplication. Shared genes are in bold lettering.
