# Supplementary material for: A comprehensive analysis of copy number variation in a Turkish dementia cohort
Source: Hum Genomics. 2021 Jul 28;15:48. doi: 10.1186/s40246-021-00346-z (PMC8317312; doi:10.1186/s40246-021-00346-z)
Supplement: Supplementary file 3 — Additional file 3: Supplementary Table 2. Likelihood ratios for CNVs reported from analysis of both Turkish dementia cohort and UK Biobank. [file 40246_2021_346_MOESM3_ESM.docx]

| **CNV** | **Turkish AD +LR** | **Turkish AD -LR** | **Turkish FTD +LR** | **Turkish FTD**  **-LR** | **Turkish MCI +LR** | **Turkish MCI**  **-LR** | **Turkish risk of FTD +LR** | **Turkish risk of FTD**  **-LR** | **Turkish dementia +LR** | **Turkish dementia -LR** | **UKBB +LR** | **UKBB  -LR** |
| --- | --- | --- | --- | --- | --- | --- | --- | --- | --- | --- | --- | --- |
| Duplication overlapping *AFG1L* and *SNX3* | . | . | . | 0.96 | . | . | . | . | . | 0.00 | . | 1.00 |
| Duplication overlapping *VWDE* | . | . | . | 0.93 | . | . | . | . | . | . | . | 1.00 |
| Duplication overlapping *HNRNPCL1* | . | 0.98 | . | . | . | . | . | . | . | . | 0.87 | 1.00 |
| Duplication overlapping *CYFIP1* and *NIPA1* | 1.16 | 1.00 | 3.41 | 0.97 | . | . | . | . | . | . | 0.91 | 1.00 |
| Duplication overlapping *ZNF804A* | . | 0.98 | . | . | . | . | . |  | . | . | . | 1.00 |
| Duplication overlapping *SNORA70B, USP34* and *XPO1* | . | 0.98 | . | . | . | . | . | . | . | . | . | 1.00 |
| Duplication overlapping *DGCR2* | . | 0.98 | . | . | . | . | . | . | . | . | 1.10 | 1.00 |
| Duplication overlapping *CNTN6* | . | . | . | . | . | 0.95 | . | . | . | . | 0.60 | 1.00 |
| Duplication overlapping *GALNTL6* | . | 0.99 | . | . | . | . | . | . | . | . | . | 1.00 |
| Deletion overlapping *MICA* | . | . | . | . | . | . | . | 0.90 | . | . | 0.98 | 1.00 |
| Duplication overlapping *MICA* | . | 0.96 | . | . | . | . | . | . | . | . | 0.56 | 1.00 |
| Duplication overlapping *MIR3691* | . | . | . | . | . | 0.95 | . | . | . | . | . | 1.00 |
| Deletion overlapping *OR13C8, OR13C5, OR13C2, OR13C9, OR13D1, NIPSNAP3A, NIPSNAP3B, ABCA1* and *LOC286367* | . | . | . | . | . | 0.95 | . | . | . | . | . | 1.00 |
| Deletion overlapping *BC039545* | . | . | . | 0.93 | . | . | . | . | . | . | . | 1.00 |

AD - Alzheimer’s disease; FTD - Frontotemporal dementia; MCI - mild cognitive impairment; +LR – positive likelihood ratio, -LR – negative likelihood ratio. A +LR above 1 indicates that a CNV increases the likelihood of the phenotype, and a -LR close to zero indicates that in the absence of a CNV, there is no effect on the likelihood of the phenotype.
