# Supplementary material for: A comprehensive analysis of copy number variation in a Turkish dementia cohort
Source: Hum Genomics. 2021 Jul 28;15:48. doi: 10.1186/s40246-021-00346-z (PMC8317312; doi:10.1186/s40246-021-00346-z)
Supplement: Supplementary file 4 — Additional file 4: Supplementary Table 3. Previously reported definitely and potentially pathogenic variants in individuals included in this cohort. [file 40246_2021_346_MOESM4_ESM.docx]

| **Variant** | **HGVS** | **# individuals within cohort** | **Reference** |
| --- | --- | --- | --- |
| TREM2 p.Tyr38Cys | ENST00000373113.3:c.113A>G | 1 | [[15]](https://paperpile.com/c/nQtoLW/kQ0N) |
| TREM2 p.Thr66Met | ENST00000373113.3:c.197C>T | 1 | [[15]](https://paperpile.com/c/nQtoLW/kQ0N) |
| PSEN2 p.Ser130Leu | ENST00000366783.3:c.389C>T | 1 | [[13]](https://paperpile.com/c/nQtoLW/iMJe) |
| PSEN2 p.Met174Val | ENST00000366783.3:c.520A>G | 2 | [[13]](https://paperpile.com/c/nQtoLW/iMJe) |
| C9orf72 expansion | ENST00000379997.3: c.-45+(GGGGCC)^n^ | 3 | [[18]](https://paperpile.com/c/nQtoLW/CWzt) |
| NOTCH3 p.Arg1231Cys | ENST00000263388.2:c.3691C>T | 2 | [[14]](https://paperpile.com/c/nQtoLW/3vgb) |
| MAPT p.Pro636Leu | ENST00000344290.5:c.1907C>T | 1 | [[18]](https://paperpile.com/c/nQtoLW/CWzt) |
| GRN p.Cys139Arg + c.-22C>T | ENST00000053867.3:c.415T>C +  ENST00000053867.3:c.-22C>T | 1 | [[18]](https://paperpile.com/c/nQtoLW/CWzt) |
