# Supplementary material for: A comprehensive analysis of copy number variation in a Turkish dementia cohort
Source: Hum Genomics. 2021 Jul 28;15:48. doi: 10.1186/s40246-021-00346-z (PMC8317312; doi:10.1186/s40246-021-00346-z)
Supplement: Supplementary file 5 — Additional file 5: Supplementary Table 4. List of neurogenes. [file 40246_2021_346_MOESM5_ESM.docx]

| **AD** | | | | | | | |
| --- | --- | --- | --- | --- | --- | --- | --- |
| *APP* | *PSEN1* | | *PSEN2* | *APOE* | *TREM2* | *CLU* | *PICALM* |
| *CR1* | *BIN1* | | *MS4A6A* | *MS4A4E* | *CD33* | *ABCA7* | *CD2AP* |
| *EPHA1* | *HLA-DRB5* | | *HLA-DRB1* | *SORL1* | *PTK2B* | *SLC24A4* | *ZCWPW1* |
| *CELF1* | *FERMT2* | | *CASS4* | *INPP5D* | *MEF2C* | *NME8* |  |
| **PD** | | | | | | | |
| *SNCA* | *PARK2/PRKN* | | *PINK1* | *PARK7/DJ-1* | *LRRK2* | *PLA2G6* | *FBXO7* |
| *VPS35* | *ATP13A2* | | *DNAJC6* | *SYNJ1* | *GBA* | *MAPT* | *RAB7L1* |
| *BST1* | *GAK* | | *ACMSD* | *STK39* | *SYT11* | *FGF20* | *STX1B* |
| *GPNMB* | *SIPA1L2* | | *INPP5F* | *MIR4697HG* | *GCH1* | *VPS13C* | *DDRGK1* |
| *MCCC1* | *SCARB2* | | *CCDC62* | *RIT2* | *SREBF1* |  |  |
| **FTD** | | | | | | | |
| *GRN* | *CHMP2B* | | *HNRNPA1* | *HNRNPA2B1* | *SQSTM1* | *OPTN* | *CHCHD10* |
| *VCP* | *SIGMAR1* | | *PRKAR1B* | *TMEM106B* | *UBQLN2* | *ATXN2* |  |
| **DLB** | | | | | | | |
| *CNTN1* | | *CYP2D6* | *EIF4G1* | *BCL7C/STX1B* | *GABRB3* | *GIGYF2* | *PRNP* |
| *SNCB* | | *SOX17* | *GBA* | *APOE* | *SNCA* |  |  |
| **Additional genes** | | | | | | | |
| *ANG* | | *CCNF* | *CSF1R* | *CTSC* | *CYLD* | *DCTN1* | *FUS* |
| *GLE1* | | *ITM2B* | *MATR3* | *NEK1* | *NOTCH3* | *PFN1* | *PNPLA6* |
| *RAB38* | | *SERPINI1* | *SOD1* | *TAF15* | *TARDBP* | *TBK1* | *TIA1* |
| *TUBA4A* | | *TYROBP* | *VAPB* |  |  |  |  |
|  |  |  |  |  |  |  |  |
