# Supplementary material for: A comprehensive analysis of copy number variation in a Turkish dementia cohort
Source: Hum Genomics. 2021 Jul 28;15:48. doi: 10.1186/s40246-021-00346-z (PMC8317312; doi:10.1186/s40246-021-00346-z)
Supplement: Supplementary file 6 — Additional file 6: Supplementary Table 5. Genes reported to overlap CNVs associated with dementia. [file 40246_2021_346_MOESM6_ESM.docx]

| **Gene(s)** | **PMID** |
| --- | --- |
| *HTRA1* | 31403081 |
| *MBD5* | 31290275 |
| *CR1** | 29675612, 27789410, 21403675 |
| *TYROBP** | 29336840 |
| *PSEN1** | 28461250, 23583593, 17594345, 12615638, 11489138, 11084029, 10854108, 10658639, 10502791, 9546792, 9197277 |
| *SULT1A3,SULT1A4* | 28374858 |
| *GRN** | 28153380, 24469240, 21677378, 23904625, 20930269, 19683260, 18479928, 18157829, 17157414 |
| *VPS13A,GNA14* | 28079535 |
| *MAPT** | 27956742, 26453547, 19263483, 23047372, 22817714 |
| *ACE* | 27862810, 27273771, 25596842, 25360660, 21770707, 21537387, 18838196, 17182125, 16970648, 16033878, 12147333, 10400221, 19452013 |
| *SDF4,NBPF10,CR1*,SLC30A3,DNAJC5G,TRIM54,BIN1*,CREB1,FAM119A,CHMP2B*,POU1F1,GBE1,EPHA6,CPNE4,EPHA5,EVC2,EVC,CRMP1,HLA-DRA* | 27662298 |
| *C4A,C4B* | 27758680 |
| *SET,JAG2,ZFPM1,APC2,CNTLN* | 26035058 |
| *C9orf72* | 25835037, 25595499 |
| *APP** | 25650802, 22545812, 21193246, 11238715 |
| *A2M* | 25574746, 14678766, 12059070, 11231028, 11121179, 11041282, 10688047, 10548657, 10449138, 10319855 |
| *SPN,QPRT,JCLN,KIF22,MAZ,PRRT2,MVP,CDIPT,PIS,SEZ6L2,ASPHD1,KCTD13,FKSG86,TMEM219,TAOK2,HIRIP3,INO80E,DOC2A,ALDOA,PPP4C,TBX6,YPEL3,GDPD3,MAPK3,CORO1A* | 25379732 |
| *CREB1,EUF3IP1,LINC00550,CHRFAM7A,GALNTL6,BX248273* | 24787912 |
| *GRID2* | 24122788 |
| *ERMP1,EVC2,EVC,CRMP1,A2BP1,ABAT,EPHA5,CDH2,EPHA6,KANK1,DMRT1,CHMP2B*,POU1F1,FLJ35024,VLDLR,MAPT*,APP** | 23752245 |
| *TESK2,CCDC163P,MMACHC,PRDX1,AKR1A1,NASP,CCDC17,RPS15AP10,GPBP1L1,TMEM69,IPP,MAST2,PIK3R3,TSPAN1,POMGNT1,LURAP1,DNAH14,LBR,VRK2,FANCL,LRP1B,KYNU,ARHGAP15,LRRTM4,SNAR-H,ZNF804A,FLJ44048,ZC3H15,ITGAV,FAM171B,ZSWIM2,CALCRL,TFPI,GULP1,DIRC1,COL3A1,COL5A2,TMEFF2,PCGEM1,ERBB4,IKZF2,SPAG16,VWC2L,PAPOLG,REL,PUS10,PEX13,KIAA1841,C2orf74,AHSA2,USP34,SNORA70B,XPO1,FAM161A, CCT4,COMMD1,TBC1D5,ROBO2,ROBO1,GBE1,CADM2,VGLL3,CHMP2B*,POU1F1,HTR1F,CGGBP1,ZNF654,C3orf38,EPHA3,TBC1D19,STIM2,PCDH7,ARAP2,FLJ16686,SNCA*,MMRN1,TMSB4XP8,KIAA1680,GRID2,KHDRBS2,LGSN,PTP4A1,PHF3,EYS,SLC25A51P1,BAI3,C6orf170,GJA1,HSF2,SERINC1,PKIB,FABP7,SMPDL3A,CLVS2,TRDN,NKAIN2,C6orf103,LOC729176,STXBP5,MAGI2,GNAI1,GNAT3,CD36,SEMA3C,HGF,CACNA2D1,PCLO,SEMA3E,SEMA3A,SEMA3D,GRM3,KIAA1324L,DMTF1,WNT2,ASZ1,CFTR,CHRM2,PTN,DGKI,SNTG1,LOC401463,BHLHE22,CYP7B1,PI15,CRISPLD1,HNF4G,ZFHX4-AS1,ZFHX4,PEX2,PKIA,ZC2HC1A,IL7,CTHRC1,SLC25A32,DCAF13,RIMS2,CDKN2B-AS1,DMRTA1,ELAVL2,TUSC1,PTPRD,TYRP1,LURAP1L,RFX3,CYLC2,SMC2,OR13F1,OR13C4,OR13C3,OR13C8,OR13C5,OR13C2,OR13C9,OR13D1,NIPSNAP3A,NIPSNAP3B,LOC286367,ABCA1,ANXA2P3,CTNNA3,LRRTM3,DNAJC12,SIRT1,HERC4,MYPN,ATOH7,PBLD,HNRNPH3,RUFY2,DNA2,SLC25A16,TET1,SNORD98,CCAR1,SOX6,C11orf58,MAML2,CCDC82,JRKL,CNTN5,SLC6A15,TSPAN19,LRRIQ1,ALX1,RASSF9,NTS,MGAT4C,LINC00442,PHF2P1,TUBA3C,ANKRD26P3,TPTE2,MPHOSPH8,PSPC1,ZMYM5,ZMYM2,POSTN,TRPC4,UFM1,FREM2,SLITRK1,SLITRK6,SLITRK5,LINC00410,MIR17HG,GPC5,GPC6,NOVA1,FOXG1,C14orf23,PRKD1,SEPT4,C17orf47,TEX14,IGBP1P2,RAD51C,PPM1E,TRIM37,FAM33A,PRR11,SMG8,GDPD1,TMPRSS15,LINC00320,NCAM2,LINC00158,MIR155HG,LINC00515, MRPL39,JAM2,ATP5J* | 23583670 |
| *ADRA2B* | 23499426 |
| *FAM63A,SOAT1,AGT,LHCGR,HLA-G,HLA-A,HLA-E,BAT1,HLA-DRA,HLA-DQB1,MAGI1,CD36,RELN,NAT2,ALDH18A1,EBF3,PICALM*,C12orf41,ALDH2,PSEN1*,NGB,SERPINF2,MYH13,MYH8,MAPT*,GALP,APP*,DOPEY2,CBS,S100B,COMT,BCR, CHRFAM7A,LCE1D,GTF2A1L,TMPRSS11E2,RP9,GRM5,ST3GAL4,CBR3,DGCR2* | 23227193 |
| *CREB1,ARL17P1,FAM119A,SDF4,NBPF10* | 23168992 |
| *SHANK3* | 22922660 |
| *CFH,ATXN1,HLA-A,MICA,HLA-DQA1,HLA-DOA,HLA-DPB1,CD36,RELN,APBA1,ABCA1,RXRA,ABCC2,PICALM*,CYP19A1,CHRNA3,MEF2A,TP53,COX10,SREBF1*,CCL3,KIF18B,DSC1,NCAM2,APP*,DOPEY2,KCNJ6,COMT,BCR,GSTT1* | 22486522 |
| *CHMP2B** | 21815258 |
| *SPAST,SLC30A6* | 21659953 |
| *OR4M1,OR4N2,OR4K2,OR4K5,OR4K1* | 21482944 |
| *CHRNA7* | 20061627 |
| *DJ-1** | 15542239 |
| *CCR5* | 15465089 |
| *LRPAP1* | 14959998 |
| *PRNP** | 11468331, 7485229, 8030960, 1363802 |
| *MIR4425,MIR5585,MIR4423,MIR7856,MIR149,MIR4792,MIR4275,MIR1271,MIR3691,MIR6836,MIR595,MIR378D2,MIR4664,MIR4476,MIR4475,MIR3689B,MIR3689D1,MIR3689F,MIR3689A,MIR3689C,MIR3689E,MIR3689D2,MIR675,MIR7155,MIR612,MIR4690,MIR4489,MIR1827,MIR6763,MIR4500,MIR4502,MIR548AR,MIR4502,MIR548AR,MIR203A,MIR203B,MIR4511,MIR4311,MIR138-2,MIR635,MIR6788,MIR7153,MIR6515,MIR5695,MIR3189,MIR1283-2,MIR527,MIR516A-1,MIR548J* | 30909216 |
| *CHRNA7,CHRFAM7A,IMMP2L,NDE1,NRXN1,ERB4,CYFIP1,NIPA1,APC2* | 30258274 |
| *CACNA1D,CACNA2D3,CACNA2D2* | 27379157 |
| *ABCA7** | 27231719 |
| *GSTT2B* | 26207597 |
| *AHR,AGR3,ATXN10,FBLN1,C1orf101,ADSS,CNTN6,EEF2K,CDR2,POLR3E,C16orf52,PDZD9,VWA3A,ZSWIM6,NDUFAF2,ELOVL7,MINA,GABRR3,CRYBG3,MSH3,RASGRF2,PTPRD,RAF1,TMEM40,MPHOSPH8,ZMYM5,PSPC1,TUBGCP5,CYFIP1,NIPA2,NIPA1,WHAMML1* | 22384383 |
| *CHRFAM7A,CSMD1,SLC35F2,HNRNPCL1,NRXN1,ERBB4,PPP2R2B,MAGI2,GRIN3A,LRRTM3,PPM1H,ADAM10,DNAJC28* | 21660214 |
| *HNRNPCL1,NRXN1,ERBB4,ATXN1,HLA-DPB1,RELN,IMMP2L,CSMD1,CHRNA7,DOPEY2,GSTT1* | 23148125 |
| *GRN*, RUNDC3A/RPIP8, SLC25A39* | 18157829 |
| *MAPT*,CTSF,ACTN3* | 28749476 |

*= genes overlapping with supplementary table 4.
